# TEsoNet: knowledge transfer in surgical phase recognition from laparoscopic sleeve gastrectomy to the laparoscopic part of Ivor–Lewis esophagectomy

**DOI:** 10.1007/s00464-023-09971-2

**Published:** 2023-03-17

**Authors:** J. A. Eckhoff, Y. Ban, G. Rosman, D. T. Müller, D. A. Hashimoto, E. Witkowski, B. Babic, D. Rus, C. Bruns, H. F. Fuchs, O. Meireles

**Affiliations:** 1 Surgical Artificial Intelligence and Innovation Laboratory, Department of Surgery, Massachusetts General Hospital, 15 Parkman Street, WAC339, Boston, MA 02114 USA; 2 Department of General, Visceral, Tumor and Transplant Surgery, University Hospital Cologne, Kerpenerstrasse 62, 50937 Cologne, Germany; 3 Computer Science and Artificial Intelligence Laboratory, Massachusetts Institute of Technology, 32 Vassar St, Cambridge, MA 02139 USA; 4 Department of Surgery, University Hospitals Cleveland Medical Center, Cleveland, OH 44106 USA; 5 Department of Surgery, Case Western Reserve School of Medicine, Cleveland, OH 44106 USA

**Keywords:** Artificial intelligence, Ivor–Lewis esophagectomy, Phase recognition, Upper gastrointestinal surgery, Transfer learning, Computer vision

## Abstract

**Background:**

Surgical phase recognition using computer vision presents an essential requirement for artificial intelligence-assisted analysis of surgical workflow. Its performance is heavily dependent on large amounts of annotated video data, which remain a limited resource, especially concerning highly specialized procedures. Knowledge transfer from common to more complex procedures can promote data efficiency. Phase recognition models trained on large, readily available datasets may be extrapolated and transferred to smaller datasets of different procedures to improve generalizability. The conditions under which transfer learning is appropriate and feasible remain to be established.

**Methods:**

We defined ten operative phases for the laparoscopic part of Ivor-Lewis Esophagectomy through expert consensus. A dataset of 40 videos was annotated accordingly. The knowledge transfer capability of an established model architecture for phase recognition (CNN + LSTM) was adapted to generate a “Transferal Esophagectomy Network” (TEsoNet) for co-training and transfer learning from laparoscopic Sleeve Gastrectomy to the laparoscopic part of Ivor-Lewis Esophagectomy, exploring different training set compositions and training weights.

**Results:**

The explored model architecture is capable of accurate phase detection in complex procedures, such as Esophagectomy, even with low quantities of training data. Knowledge transfer between two upper gastrointestinal procedures is feasible and achieves reasonable accuracy with respect to operative phases with high procedural overlap.

**Conclusion:**

Robust phase recognition models can achieve reasonable yet phase-specific accuracy through transfer learning and co-training between two related procedures, even when exposed to small amounts of training data of the target procedure. Further exploration is required to determine appropriate data amounts, key characteristics of the training procedure and temporal annotation methods required for successful transferal phase recognition. Transfer learning across different procedures addressing small datasets may increase data efficiency. Finally, to enable the surgical application of AI for intraoperative risk mitigation, coverage of rare, specialized procedures needs to be explored.

**Graphical abstract:**

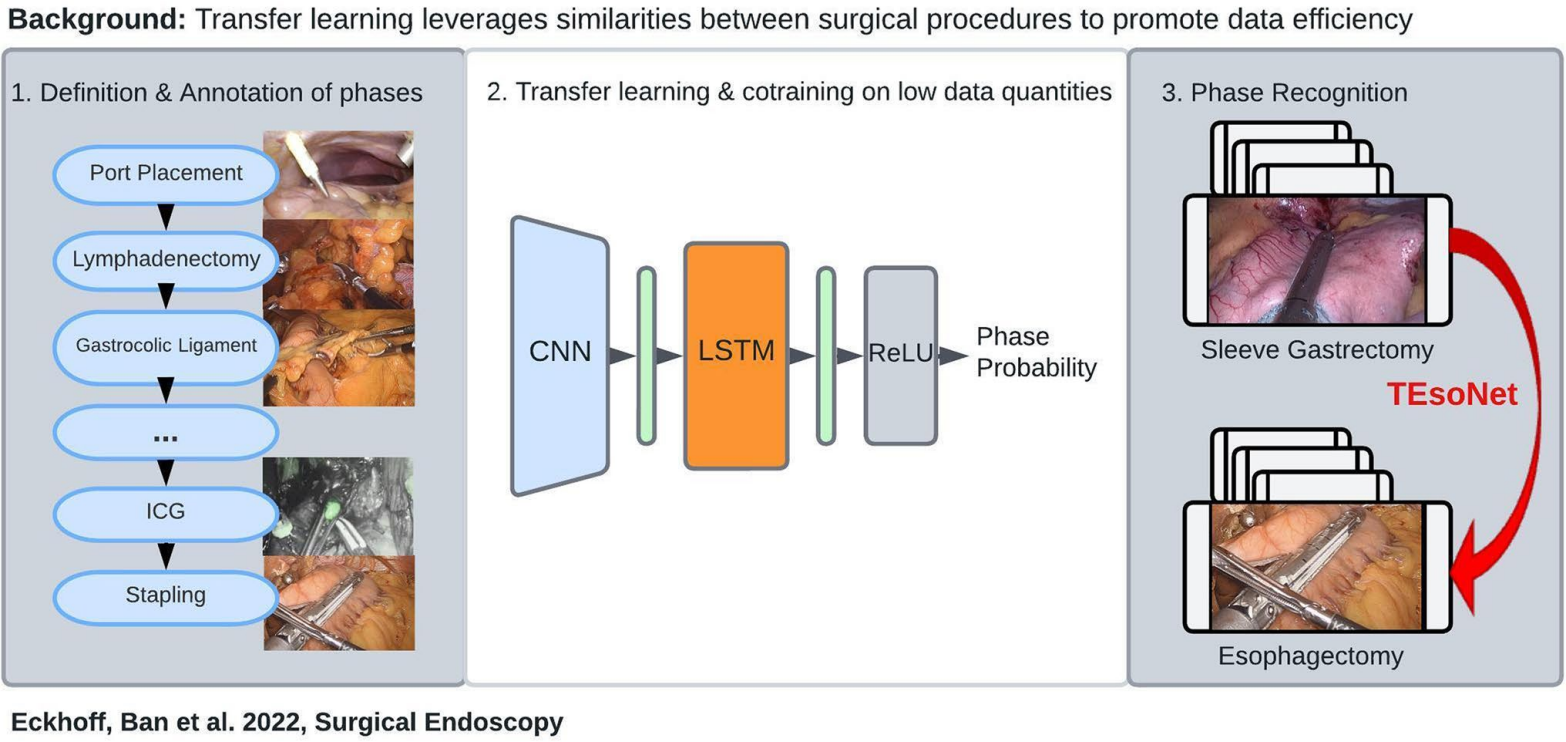

Through comprehension of spatial, temporal as well as conceptual aspects of surgical workflow, artificial intelligence (AI) holds the potential to change minimally invasive surgery. The merits of computer vision (CV) and machine learning (ML), subfields of AI, range from intraoperative decision support, and improvement of surgical teaching to prediction of operative complications and reduction of surgical risk. Current applications include temporal analysis, such as phase detection and prediction [1–4] and spatial analysis, including instrument segmentation [5–7] and the detection of ideal dissection areas [8]. In parallel more conceptual approaches, such as the comprehension of surgical notions like the Critical View of Safety and Action Triplets, aim to understand and mimic surgical thinking [9, 10]. Particularly the temporal comprehension of surgical workflow serves as translational knowledge in the field of surgical AI [11]. Surgical phase detection and prediction promises the potential to detect deviations from a regular operative course and foresee complications before they arise [12]. Currently, laparoscopic cholecystectomy serves as the benchmark for surgical AI due to its high prevalence, stable view, and standardized procedural sequence. Successful recognition of operative phases has also been demonstrated in Sleeve Gastrectomy [4], Perioral Endoscopic Myotomy (POEM) [3] and Sigmoidectomy [13]. However, in order to have a significant impact on patient safety, AI should be applicable to high-stake surgical procedures associated with notable risk.

Traditional ML models undergo training on labeled or “annotated” imaging data, learn to extract target features, and subsequently detect these features in test data. Their performance is measured in concordance with the ground truth labels of expert annotators. To achieve good model performance large quantities of data, and adequate annotations are required to train the models. Furthermore, diversity within the data and coverage of rare events ensures generalizability of the model and applicability to a wide range of cases. This stands in contrast to the lack of annotated surgical video data capturing high-stake, rare procedures of immense clinical significance, such as Ivor-Lewis Esophagectomy for Esophageal cancer. With less than 2000 cases performed in the US annually [14], a major complication rate of 59% [15] and mortality rates around 3.1% [16], Esophagectomy is one of the most difficult oncological operations worldwide. However, the attempt to leverage AI for risk mitigation in Esophagectomy is met by major obstacles. Due to its highly complex workflow, both during the abdominal and thoracic part of the operation, Esophagectomy does not lend itself to exploration through AI algorithms tailored to highly standardized procedures. The relative paucity of imaging data of Esophagectomy cases, compared to previously investigated procedures, force the community to look creatively at related procedures and investigate similar target features to address. Moreover, the definition of target features and appropriate annotation guidelines for such procedures require careful consideration to be simultaneously clinically relevant as well as applicable to ML algorithms [17].

Surgical residents learn to transfer acquired practical skills to novel situations, as they gradually move from performing individual steps of a procedure, to completing entire procedures as the primary operating surgeon. Analogous to this cognitive phenomenon, transfer learning (TL) refers to ML models leveraging information from source datasets, to optimize their performance on new target datasets with similar characteristics. This enables algorithms to make inferences about new datasets with small data quantities for training [18]. As AI aims to mimic human intelligence, it appears plausible to exploit such adaptive learning techniques to augment algorithmic performance by exposing the models to operations with procedural overlap and common visual features. TL between related procedures may present a solution to the problem of limited surgical video data availability and move AI closer to real-time deployment the operating room. Prior work using TL for surgical step recognition has demonstrated accurate recognition of general surgical tasks, such as adhesiolysis, dissection and inspection in four different procedures [13]. However, the specific, contextual workflow and interdependencies between individual phases of an operation offers more information. Much like chapters in a book the individual phases, further divided into steps, actions and tasks, offer an overall narrative of the specific goal of a surgical procedure. In order for ML models to fully comprehend surgical thinking, they should be able to conceptualize this semantic information and relations between phases and be tailored to extrapolate knowledge, similar to surgical trainees.

In this study we explore TL for phase recognition on laparoscopic part of Ivor-Lewis (IL) Esophagectomy. With our “Transfer Esophagectomy Network” (“TEsoNet”), we explore the capability of an established model architecture for phase recognition (a Convolutional Neural Network (CNN) and a Long Short Term Memory (LSTM) Network) to transfer knowledge between two related upper gastrointestinal procedures. We aim to overcome data shortage and promote data efficiency by training a model on an existing dataset of laparoscopic Sleeve Gastrectomy (source data) and evaluate its performance on the laparoscopic part of IL-Esophagectomy (target data). To determine ideal prerequisites for successful TL between the two procedures, we investigate different proportions of target and source data in model training and look into the effect of co-training on both procedures. Moving beyond recognition of general surgical actions, we will explore the conditions under which knowledge transfer is feasible with regard to specific, semantically dependent, clinically relevant phases. For this purpose we divided the laparoscopic part of IL-Esophagectomy into three distinct steps with clinically meaningful goals: (1) the lymphadenectomy, (2) the preparation of the Gastric Conduit, and (3) the preparation of the diaphragmatic Hiatus. More specifically, we are the first to define and map out concise procedural phases within these steps, in concordance with the steps described by Fuchs et al. [19]. This clear definition of annotations of surgical phases is paramount to the development of ML capable of mimicking surgical intelligence. Overall our objective is to evaluate the feasibility of knowledge transfer between two different laparoscopic upper gastrointestinal procedures, analogous to the learning process of surgical residents. Beyond that, we investigate the data requirements and conditions required for transfer learning. The overall methodology includes (1) the granular phase definition and annotation of the laparoscopic part of IL-Esophagectomy through expert consensus, (2) the validation of a CNN-LSTM Network for phase recognition on Sleeve Gastrectomy and Esophagectomy and (3) exploration of the impact of training data quantity and quality on phase recognition accuracy in transfer learning and co-training.

## Materials and methods

### Video data

The video data of 80 laparoscopic Sleeve Gastrectomy cases were recorded at the Department of Surgery at Massachusetts General Hospital, Boston, between October 2015 and September 2018 (Institutional Review Board Protocol No. 2015P001161/PHS). The video data of 40 cases of the laparoscopic part of IL-Esophagectomy was collected at the University Hospital Cologne, Germany, between June 2021 and April 2022 (Ethics Commission Review no. 21–1132). The video data was recorded in high definition with a resolution of 1920 × 1080 pixels (Esophagectomy) and 1280 × 720 pixels (Sleeve Gastrectomy), with an aspect ratio of 16:9. Both institutions used the MediCapture USB 200 and MediCapture MVR Pro HD (MediCapture Plymouth, PA, USA) to record the video data. Inclusion criteria for subjects were (1) undergoing laparoscopic sleeve gastrectomy for bariatric weight loss surgery or the laparoscopic part of IL-Esophagectomy for esophageal cancer, (2) minimum age > 18 years, (3) sufficient video quality (minimum resolution of 720p, sufficient camera cleanliness without obscuration of the camera), (4) sufficient video completeness (recording of the majority of defined operative phases, excluding port placement and liver retraction).

### Phase definition

**Fig. 1:**
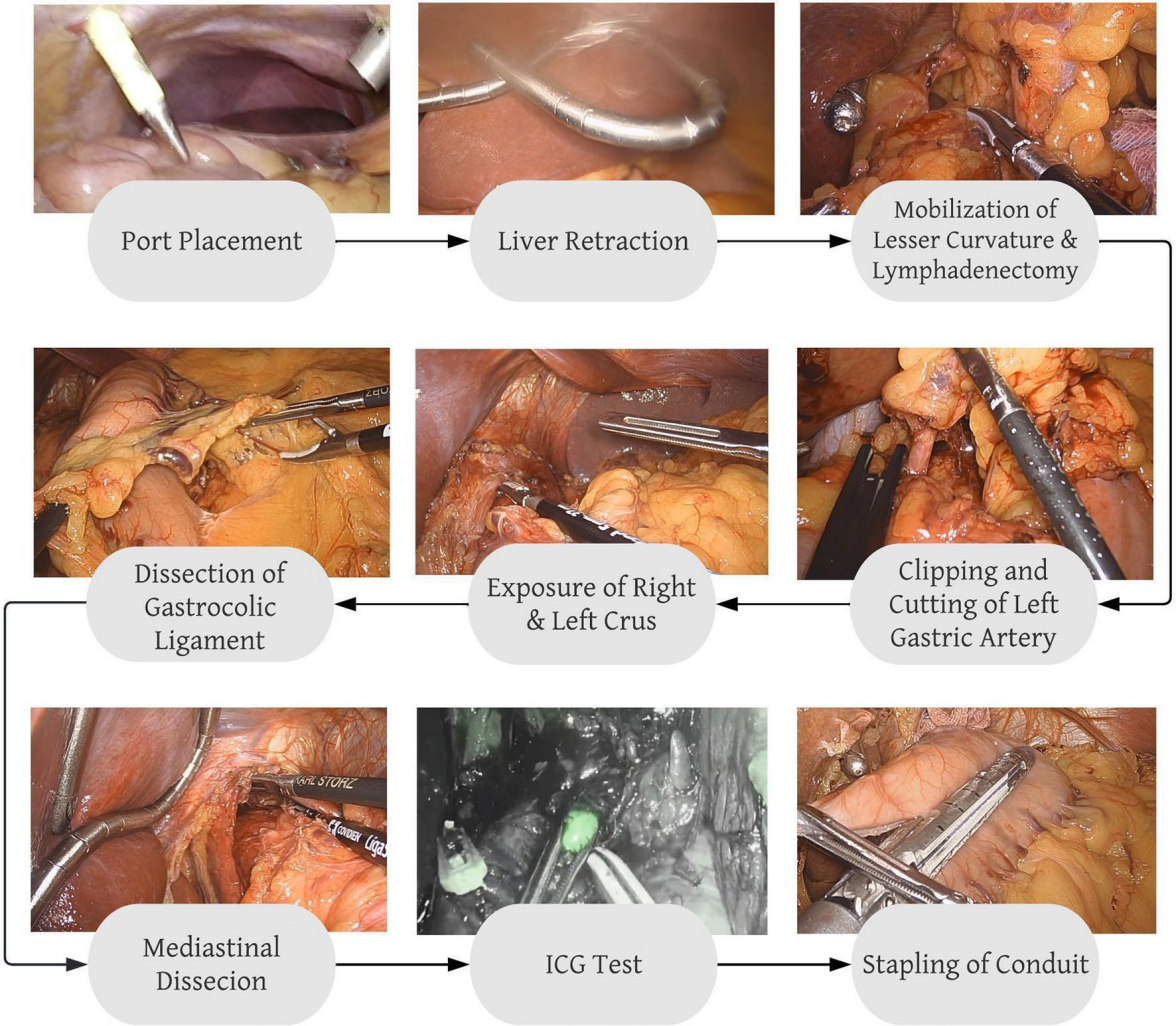
Overview of the surgical workflow and definition of phases in the laparoscopic part of Ivor–Lewis esophagectomy

The phase definitions for laparoscopic Sleeve Gastrectomy were used as established by Hashimoto et al. [4]. To define the procedural phases of the laparoscopic part of IL- Esophagectomy, an expert panel of three board-certified upper-GI surgeons from Massachusetts General Hospital, Boston, USA, and University Hospital Cologne, Germany were consulted. In order to achieve clinically meaningful, yet CV-compatible phase definitions, the SAGES annotation framework [17] served as a guideline. The resulting phase definitions were: (1) Port Placement (2) Liver Retraction (3) Dissection of the Gastrohepatic Ligament (4) Clipping and Division of the Left Gastric Artery (5) Hiatal Dissection (6) Dissection of the Gastrocolic Ligament (7) ICG perfusion check (8) Stapling of the Gastric Conduit (9) Mediastinal Dissection and (10) Inspection. A detailed description of the individual phase definitions is given in Table 1. Figure 1 shows the procedural workflow during the laparoscopic part of IL-Esophagectomy, as captured in our phase definitions. The two procedures were compared in terms of: (i) procedural similarities and differences, (ii) the temporal evolvement and phase transitions, and (iii) the visual characteristics specific to individual phases.

**Table 1:** Overview of phase definitions in the laparoscopic part of Ivor-Lewis Esophagectomy

Esophagectomy phases	Start point	Endpoint	Description
Port placement	When the tip of the introducer violates the peritoneum	Upon removal of the introducer	Port placement in a standardized fashion for Esophagectomy
Liver retraction	Introduction of Liver hook	Upon full retraction of the liver	
Dissection of gastrohepatic ligament	Upon first grasping the Gastrohepatic ligament with the grasper	When working instruments let go of Gastrohepatic ligament / upon initiation of different phase	Including complete lymphadenectomy along the lesser curvature and exposure and preparation of the left gastric artery and retro gastric preparation close to hiatus
Clipping + division of left gastric artery	Upon introduction of the clip applier	When the left gastric artery is fully divided	
Hiatal dissection	Upon first violation of the muscular structures of the Diaphragm	Upon initiation of different phase / when the instrument moves beyond the Diaphragm to proceed to mediastinal dissection	Including the dissection of the left and right Crus of the esophageal hiatus
Dissection of gastrocolic	Upon the first grasp of the Gastrocolic ligament	Upon initiation of different phase	Includes full separation of Gastrocolic and Gastrosplenic ligament and mobilization of the Duodenum
Stapling	Upon the appearance of the stapler	Upon final removal of the stapler	
Mediastinal dissection	When the instrument starts to dissect above the Diaphragm	Upon initiation of a different phase/when the camera and instrument position moves away from hiatus	Including lymphadenectomy above the Diaphragm, preparation of conduit pull through
ICG check	Upon first light change from white light to fluorescence	When fluorescence is switched to white light	Inspection of conduit and assessment of planned lymphadenectomy under fluorescent light
Inspection	When no Instrument is actively dissecting for > 10 s	When dissection action is resumed /upon initiation of a different phase	Includes final inspection of the stapling line and white light inspections in between phases

### Pre-processing and annotation of surgical video data

To comply with data privacy regulations, the data was first deidentified, including removal of all metadata. Metadata included all patient data, the date, time and location of the operation as well as information about operating staff. Preprocessing was performed using a self-coded python script with ffmpeg package 4.1 (www.ffmpeg.org). The annotation of operative videos was performed by surgical residents (PGY 3 and above). The software Anvil Video Annotation Research Tool by Michael Kipp (http://www.anvil-software.org) was used for annotation of all of the Sleeve Gastrectomy videos. The Esophagectomy videos (*n* = 20) were annotated in.csv spreadsheet files. All annotations were converted to protobuff format to facilitate the model data loading.

### Model architecture

**Fig. 2:**
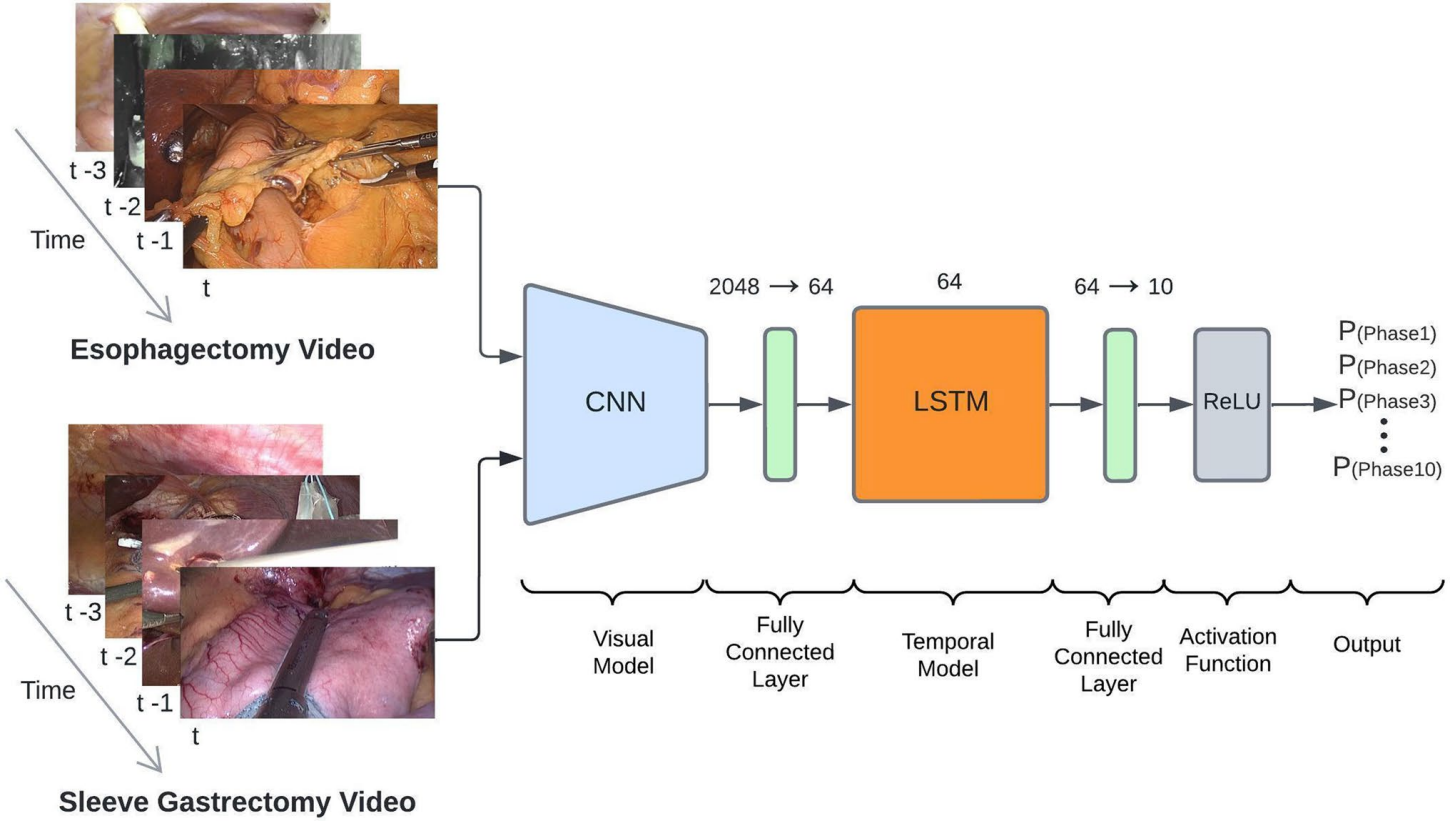
Overview of the model architecture of the Transfer Esophagectomy Network (TEsoNet): The individual video frames from laparoscopic Sleeve Gastrectomy and the laparoscopic part of Ivor-Lewis Esophagectomy are loaded in to a convolutional neural network (CNN). A following fully connected layer transforms the output for loading into a long short term memory (LSTM) model. Additional transformation through a second fully connected layer reduces the output to 10 classes before undergoing a rectified linear activation function (ReLU). The output is a probability for each individual class (phase)

The proposed TEsoNet model aims at transfer learning from the source procedure (Sleeve Gastrectomy) to the target procedure (the laparoscopic part of IL-Esophagectomy) [3]. The backbone of the utilized ML model was built by an established Convolutional Neural Network (CNN) for visual feature recognition, augmented by and a Long Short Term Memory (LSTM) model for temporal feature recognition. To account for the different feature distributions across the two procedures the last fully connected layer of the CNN was altered (see Fig. 2). The subsequent LSTM adds contextual information from past video frames to current frames. The final fully connected layer serves as a classification layer, providing probabilities for each of the 10 phases of the target procedure. In order to integrate the datasets from two procedures with different temporal structures, the phases were mapped into a single set of phases and fused for suitability to one classification task (surgical phase recognition). The data loading was performed in a 1:1 ratio (equal video segments of Sleeve Gastrectomy to Esophagectomy but derived from different quantities of videos). A combined data loader is used to load the video data from both procedures into the model. The consecutive video was broken down into short video segments of 8 s at a framerate of 1 frame per second (fps). We used the focal loss[20] for training with a learning rate of 10^–4^. The model was trained for 30 epochs (See Table 2).

**Table 2:** Comparison of phases in laparoscopic sleeve gastrectomy and the laparoscopic part of IL-Esophagectomy

Esophagectomy phases	Sleeve gastrectomy phases	Similarities	Differences	Challenges
Port placement	Port placement	Visually indifferent, an occurrence at the beginning of the video	Position of optical port/instrument manufacturer	
Liver retraction	Liver retraction	Visually indifferent, an occurrence at the beginning of the video	Instrument manufacturer	
N/A	Liver Biopsy	N/A	N/A	
Dissection of gastrohepatic Ligament	N/A	N/A	N/A	Few visual similarities between gastrohepatic and gastrocolic ligament
Clipping + division of left gastric artery	N/A	N/A	N/A	Mostly no alternative Clipping actions in Sleeve Gastrectomy
Hiatal dissection	N/A	N/A	N/A	A very short segment of dissection of the left Crus within phase “Gastrocolic Ligament Dissection” in Sleeve Gastrectomy
Dissection of gastrocolic ligaMent	Dissection gastrocolic ligament	Same anatomic location and visual surroundings, similar durations	Dissection closer to the gastric conduit in Esophagectomy / different instruments: Harmonic vs	
Stapling	Stapling stomach	Same anatomic location and visual surroundings, similar durations, number of stapling rows	Stapling side of the stomach, stapling angle,	Different stapling side of the stomach and stapling angle
N/A	Bagging specimen	N/A	N/A	No bagging in esophagectomy, hence no results shown
Mediastinal dissection	N/A	N/A	N/A	Not existent in Sleeve gastrectomy
ICG check	N/A	N/A	N/A	No fluorescent inspection in Sleeve gastrectomy by visually very distinct phase
Final inspection	Final inspection	No active instruments/same anatomic operative field	A different angle of inspection due to different port position	Occurs at the end of Sleeve gastrectomy vs. very intermittent during esophagectomy

### Model training and experimental setup

Five experiments were conducted to determine the ideal amount of data from the target procedure and the optimal ratio between target and source procedure within the training data for reliable phase recognition through TEsoNet. All experiments were performed in 3 randomly composed training splits. In experiment 1, TEsoNet was trained and tested on sleeve gastrectomy videos (train *n* = 60/test *n* = 20) in order to validate our previous results [4]. In experiment 2 TEsoNet’s performance of a Esophagectomy was evaluated (train *n* = 5, 10, 20, 30 consecutively/test *n* = 10). Experiment 3 was conducted training entirely on Sleeve Gastrectomy (*n* = 60) and testing on Esophagectomy (*n* = 10) to investigate pure TL. Experiments 4 and 5 investigated co-training on different quantities of target and source procedures in the training data. The detailed training data composition can be found in Table 3.

**Table 3:** Training/Testing split and experimental setup

Experiment	Training dataset	Validation dataset
1. Validation on sleeve gastrectomy (Source)	Sleeve (*n* = 60)	Sleeve (*n* = 20)
2. Validation on the laparoscopic part of IL-Esophagectomy (Target)	A. Eso (*n* = 5) B. Eso (*n* = 10) C. Eso (*n* = 20) D. Eso (*n* = 30)	Eso (*n* = 10)
3. Transfer learning	Sleeve (*n* = 60)	Eso (*n* = 10)
4. Co-training dependency on target procedure	A. Eso (*n* = 5) + Sleeve (*n* = 60) B. Eso (*n* = 10) + Sleeve (*n* = 60) C. Eso (*n* = 20) + Sleeve (*n* = 60) D. Eso (*n* = 30) + Sleeve (*n* = 60)	Eso (*n* = 10)
5. Co-training dependency on source procedure	A. Eso (*n* = 20) + Sleeve (*n* = 15) B. Eso (*n* = 20) + Sleeve (*n* = 30) C. Eso (*n* = 20) + Sleeve (*n* = 45) D. See experiment 4.C	Eso (*n* = 10)

## Results

Model performance was assessed by phase recognition accuracy in percent (%). Accuracy was defined as the sum of true negatives and true positives divided by all obtained values [(TN + TP) / (TN + TP + FN + FP)]. In a classification task, this represents the number of correct predictions estimated by the model out of the total number of samples.

### Temporal annotations and feature comparison

**Fig. 3:**
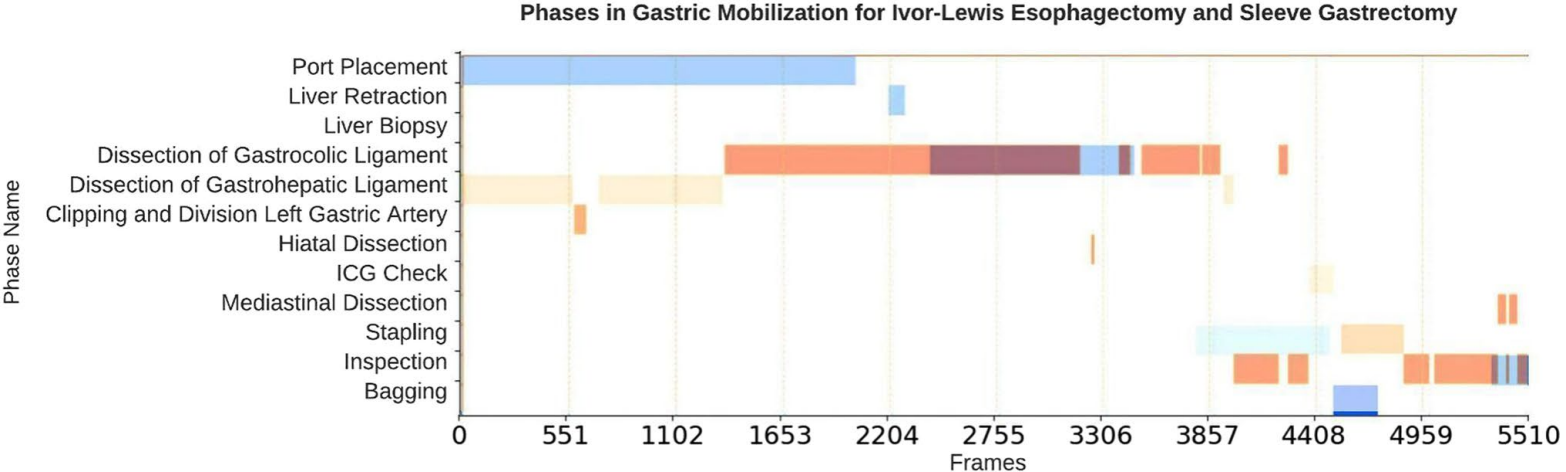
An example of the overlap in phase transitions between the laparoscopic part of Ivor-Lewis Esophagectomy (orange) and laparoscopic Sleeve Gastrectomy (blue) (Color figure online)

**Fig. 4:**
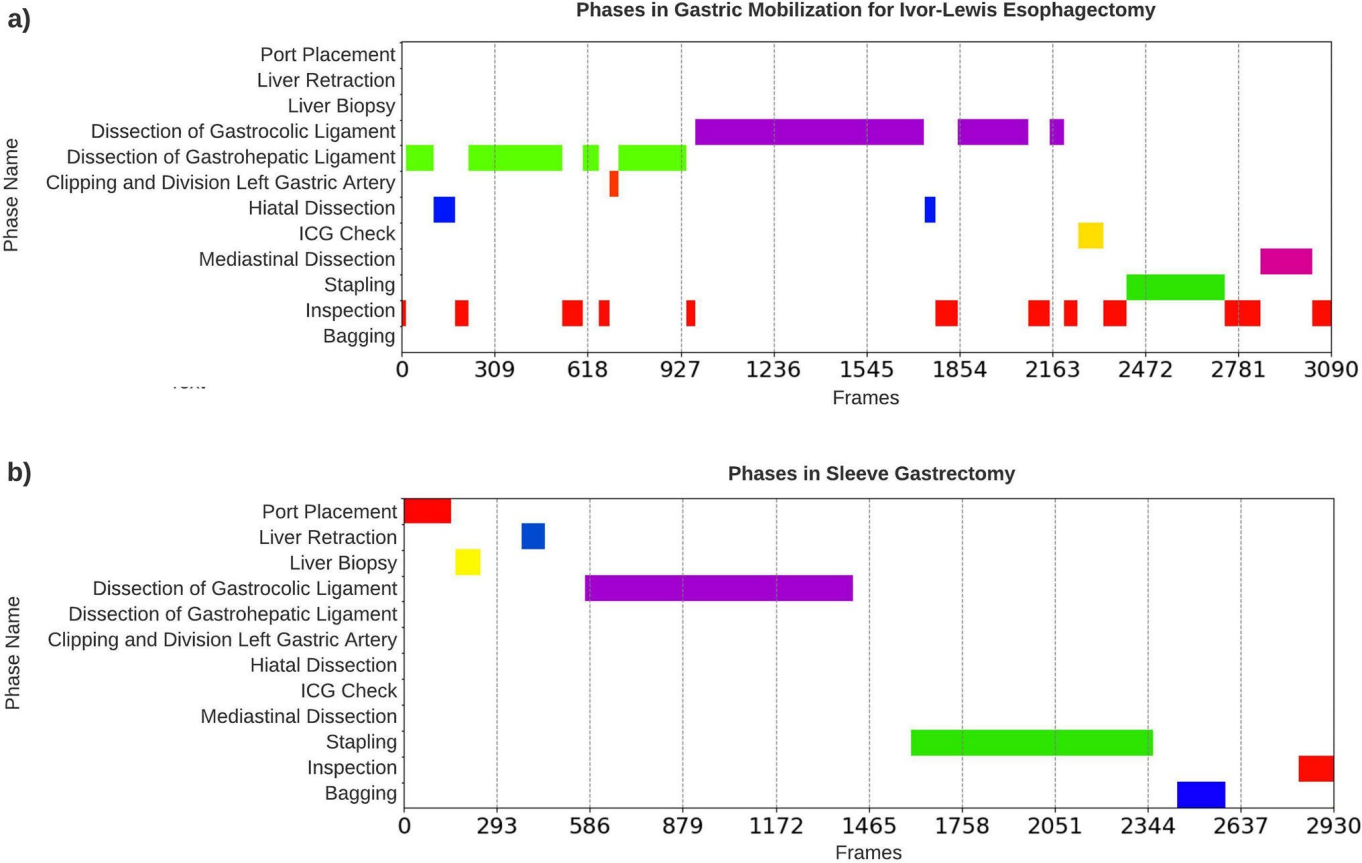
An illustration of the phases and transitions in **a** the laparoscopic part of Ivor–Lewis Esophagectomy and **b** laparoscopic Sleeve Gastrectomy. Note the linear progression in the Sleeve Gastrectomy workflow, observed by the diagonal pattern in plot (**b**). Ivor–Lewis Esophagectomy displays shorter phases and more frequent transitions

There was a significant heterogeneity between the two procedures with respect to duration of the individual phases and temporal progression of the procedure. Figure 3 and 4 compares the temporal structure of Sleeve Gastrectomy and Esophagectomy. The total duration of the laparoscopic Sleeve Gastrectomy cases was 123.49 ± 20.00 min, whereas for Esophagectomy cases it was 136.54 ± 17.14 min. In Esophagectomy the phase duration ranged from a mean ± STD of 0.1 ± 0.3 min to 46.52 ± 38.34 min. In contrast, the longest phase in Sleeve Gastrectomy was 19.56 ± 9.21 min. The Esophagectomy cases show significantly more transitions between phases (23.98 ± 5.92) and overall a less linear workflow. In Sleeve Gastrectomy there are fewer transitions between the phases (5.55 ± 1.04). The procedural overlap, visual similarities, and differences between the phases of the two procedures are summarized in Table 2. The individual phase durations in Sleeve Gastrectomy and Esophagectomy are summarized in Tables 4 and 5.

**Table 4:** Duration of operative phases in the laparoscopic part of IL-Esophagectomy

Phase	Duration mean	Duration STD	Duration min	Duration max
Port placement	0:00:10	0:00:30	0:00:00	0:02:45
Liver retraction	0:00:15	0:00:45	0:00:00	0:04:04
Dissection Of gastrohepatic ligament	0:19:58	0:18:13	0:02:08	1:08:30
Clipping + division of left gastric artery	0:01:05	0:00:44	0:00:00	0:04:20
Hiatal dissection	0:05:19	0:04:46	0:00:00	0:18:48
Dissection of gastrocolic ligament	0:46:52	0:38:34	0:01:30	2:33:05
Stapling	0:04:48	0:02:20	0:00:00	0:13:10
Mediastinal dissection	0:01:53	0:02:00	0:00:00	0:10:51
ICG	0:01:42	0:01:41	0:00:02	0:08:17
Inspection	0:08:32	0:08:34	0:00:56	0:49:03
Overall duration of the video	1:16:54	0:17:14	0:49:08	2:00:55

**Table 5:** Duration of operative phases in laparoscopic sleeve gastrectomy

Phase	Duration mean	Duration STD	Duration min	Duration max
Port placement	0:04:09	0:03:55	0:00:00	0:33:54
Liver retraction	0:00:39	0:00:33	0:00:00	0:03:58
Liver biopsy	0:01:10	0:00:37	0:00:00	0:04:34
Dissection Of gastrocolic ligament	0:19:56	0:09:21	0:00:00	0:49:32
Stapling	0:14:41	0:06:21	0:00:00	0:39:59
Bagging	0:03:17	0:02:00	0:00:00	0:10:35
Inspection	0:01:47	0:01:52	0:00:00	0:09:07
Overall duration of the video	1:03:49	0:20:17	0:27:14	2:08:24

### Experimental results: single-procedure datasets and transfer learning (Experiment 1–3)

In experiment 1, TEsoNet demonstrated good overall accuracy of 87.7 ± 7% on phase recognition in Sleeve Gastrectomy. This shows that the previous phase recognition results of SleeveNet [4] are reproducible within the expected dataset variability. By training and validating the model on Esophagectomy in experiment 2, the model proved applicable to new, complex procedures. When training on as little as five videos the overall accuracy was 36.3 ± 30.7% and could be increased to 49.7 ± 29.8% by training on 30 videos. Notably, phases with low prevalence in the dataset, such as “Port Placement” and “Liver Retraction” resulted in significantly lower accuracy (maximum accuracy of 6.8 ± 2.9% and 7.3 ± 3.6%, respectively). In contrast, long and omnipresent phases, like the “Dissection of the Gastrocolic Ligament” achieved very high accuracy (91.8 ± 4.7%). In experiment 3 we evaluated TL between the two procedures by training the model entirely on Sleeve Gastrectomy and evaluating its performance on Esophagectomy. The results show that TEsoNet achieves a high model accuracy on phases with high procedural overlap between the procedures (“Dissection of the Gastrocolic Ligament” 60.7 ± 11.3%, “Port Placement” 68.2 ± 13.5% and “Liver Retraction” 37 ± 40%). Despite visual differences in the “Stapling” phase, resulting from stapling on different curvatures of the gastric conduit, the model performance of recognizing the phase is 68.5 ± 7%. This can be explained by spatial recognition of the stapler by the CNN or correct categorization of the temporal occurrence of the phase after the “Dissection of the Gastrocolic Ligament” by the LSTM. Due to the complete absence of other phases within the Sleeve Gastrectomy training data, such as “Dissection of Gastrohepatic Ligament”, knowledge transfer did not occur for these phases. Furthermore, the low accuracy in absent phases contributes to a low overall model accuracy of 23.4 ± 31.4%, whereas the accuracy across the four overlapping phases is 58.6 ± 14.8%. The results of experiments 1, 2, and 3 are given in Table 6.

**Table 6:** Model accuracy per phase for experiments 1, 2 and 3

Experiment	1	2A	2B	2C	2D	3
Training	60 Sleeve	5 Eso	10 Eso	20 Eso	30 Eso	60 Sleeve
Testing	20 Sleeve	10 Eso	10 Eso	10 Eso	10 Eso	10 Eso
Port placement	0.9147	0.0037	0.0093	0.0395	0.0684	0.6824
Liver retraction	0.8078	0.0000	0.0000	0.0042	0.0737	0.3711
Gastrocolic ligament dissection	0.9127	0.8100	0.8857	0.8954	0.9185	0.6076
Stapling	0.9660	0.6230	0.6715	0.6972	0.7276	0.6854
Inspection	0.7620	0.3224	0.4333	0.4663	0.5034	0.0015
ICG	x	0.7766	0.7431	0.7306	0.8149	0.0000
Gastrohepatic ligament dissection	x	0.4861	0.6601	0.7774	0.7892	0.0000
Clipping + division of left gastric artery	x	0.1318	0.1726	0.2597	0.2743	0.0000
Hiatal dissection	x	0.2187	0.2822	0.2884	0.3910	0.0000
Mediastinal dissection	x	0.2616	0.2818	0.3460	0.4137	0.0000
Liver biopsy	0.8979	x	x	x	x	x
Bagging	0.8832	x	x	x	x	x
Overall	0.8777	0.3634	0.4140	0.4505	0.4975	0.2348
Standard deviation	0.0696	0.2983	0.3138	0.3138	0.3070	0.3146

### Experimental results: co-training on target and source procedure (Experiment 4–5)

**Fig. 5:**
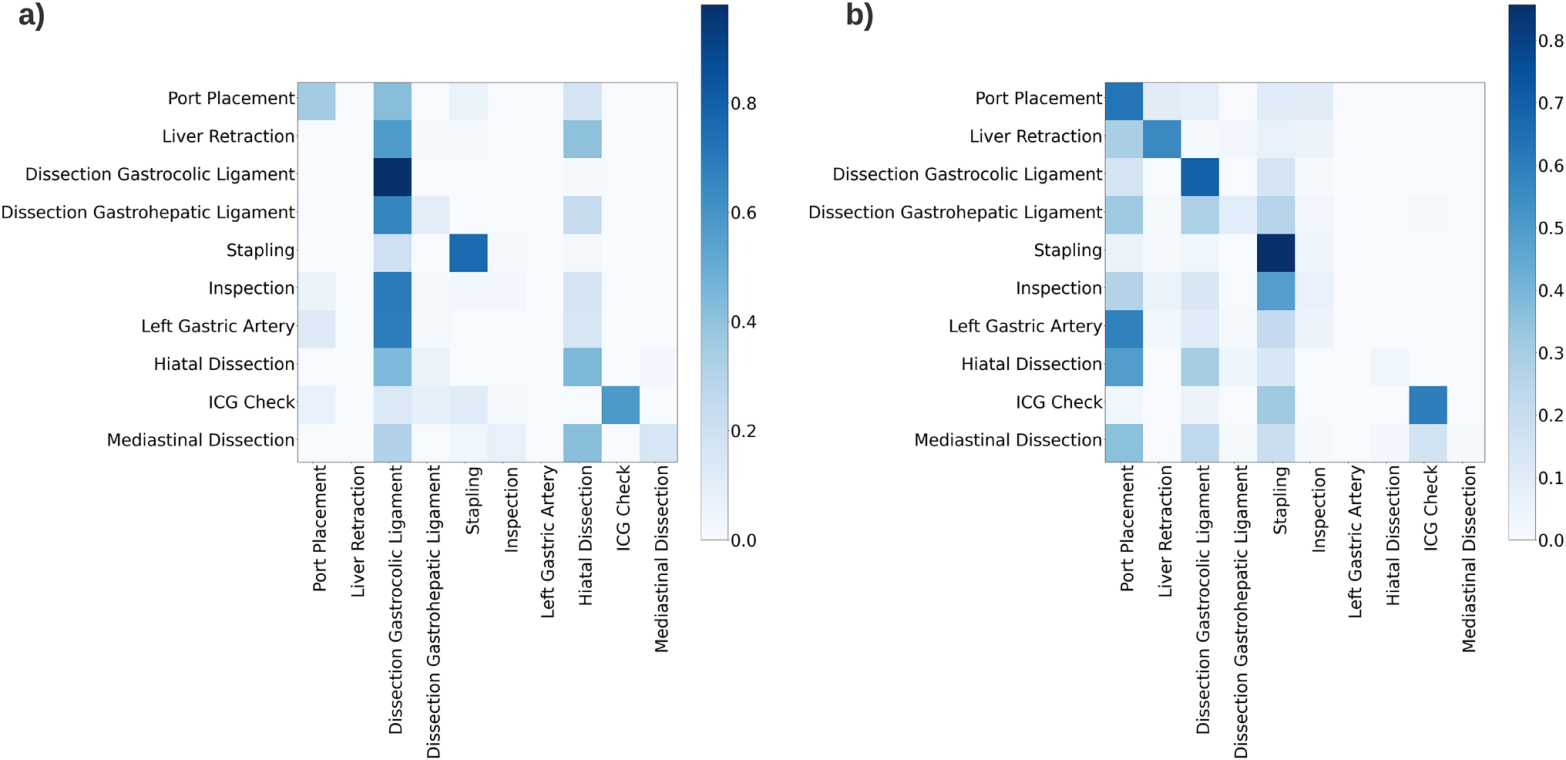
The heatmap illustrates the confusion matrix of experiment 4.D and 5.A. This represents the performance of the Transfer Esophagectomy Network (TEsoNet) on the classification task when training on different quantities of the source procedure (laparoscpic Sleeve Gastrectomy). Higher contrast demonstrates the overlap between model prediction and annotated ground truth and shows higher phase recognition accuracy

The effect on phase recognition accuracy when co-training on both procedures, using different quantities of source and target data, were explored in experiments 4 and 5. The complete results are given in Table 7. In experiment 4 co-training was performed using 60 Sleeve Gastrectomy videos and varying numbers of Esophagectomy. The results show that model performance depends heavily on the quantities of the target procedure within the training data. The overall accuracy of the model ranged from 24.5 ± 21%, when trained on five Esophagectomy videos, and increased up to 36.1 ± 30% accuracy when training was conducted using 30 Esophagectomy videos. It is notable that adding Sleeve Gastrectomy to the training data significantly improves accuracy on phases with procedural overlap (“Port placement”, “Liver Retraction”, “Dissection of Gastrocolic Ligament” and “Stapling”), compared to merely training the model on Esophagectomy. In experiment 5, the effect of different quantities of source procedure in the training data becomes apparent. In particular, the addition of Sleeve Gastrectomy in model training led to an increase in accuracy for phases with low prevalence in the Esophagectomy data (“Port Placement” vs. and “Liver Retraction”). The heatmap in Fig. 5 illustrates the confusion matrix of experiments 4.D and 5.A, representing the performance of TEsoNet on the classification task when training on different quantities of the source procedure. Higher contrast represents the overlap between model prediction and annotated ground truth, hence higher phase recognition accuracy. Overall co-training on both procedures shows that training on moderate amounts of related procedures yields the highest overall model accuracy (Experiment 5B: model accuracy of 40.8 ± 32.6%). It is important to balance augmentation of training with related procedures for improved model supervision and limiting the corruption of model performance due to `noise’ from unrelated phases.

**Table 7:** Model accuracy per phase for experiments 4 and 5

Experiment	4A	4B	4C	4D	5A	5B	5C
Training	5 Eso. + 60 Sleeve	10 Eso. + 60 Sleeve	20 Eso. + 60 Sleeve	30 Eso. + 60 Sleeve	20 Eso. + 15 Sleeve	20 Eso. + 30 Sleeve	20 Eso. + 45 Sleeve
Testing	10 Eso	10 Eso	10 Eso	10 Eso	10 Eso	10 Eso	10 Eso
Port placement	0.5628	0.5627	0.7204	0.3558	0.7090	0.7673	0.5229
Liver retraction	0.3976	0.2262	0.2324	0.2204	0.4047	0.4493	0.3529
Gastrocolic ligament dissection	0.1349	0.8950	0.8722	0.9595	0.8273	0.8998	0.8797
Stapling	0.4917	0.7612	0.5275	0.7605	0.8214	0.8270	0.8081
Inspection	0.0422	0.1209	0.1598	0.1347	0.1020	0.1674	0.2541
ICG	0.3147	0.3131	0.5853	0.5695	0.2364	0.4899	0.5790
Gastrohepatic ligament dissection	0.2449	0.1406	0.1941	0.1360	0.1020	0.2090	0.1502
Clipping + division of left gastric artery	0.0000	0.0943	0.0945	0.1148	0.0199	0.0562	0.1016
Hiatal dissection		0.0422	0.0708	0.2167	0.0133	0.0573	0.0624
Mediastinal dissection	0.0159	0.0366	0.0776	0.1503	0.0090	0.1596	0.1714
Liver biopsy	x	x	x	x	x	x	x
Bagging	x	x	x	x	x	x	x
Overall	0.2450	0.3193	0.3535	0.3618	0.3245	0.4083	0.3882
Standard deviation	0.2109	0.3115	0.2960	0.2998	0.3416	0.3268	0.2951

## Discussion

TEsoNet achieves reasonable phase recognition accuracy in most phases of the laparoscopic part of IL-Esophagectomy. Particularly good phase recognition was achieved with respect to phases with high procedural overlap between target and source procedure. This shows that TL for surgical phase recognition may be applied analogously to human learning experiences. Whilst low data abundance and diversity remain a significant challenge for surgical AI, TL and co-training have the potential to augment ML model performance selectively [18]. Furthermore TL may facilitate leveraging surgical AI for risk mitigation in high-stake operations with a direct impact on patient safety. Particularly complex surgical procedures, like Esophagectomy, are highly underrepresented as few attempts have been made to investigate these procedures using CV. This may be linked to data characteristics, which are unfavorable for ML-based analysis. ML algorithms are largely based on probabilistic identification of patterns within the data [21, 22]. Therefore, the disrupted temporal structures, rather than linear workflows, as well as short phase durations and frequent transitions, present a challenging endeavor for phase recognition. We show that TL demonstrates limitations with regard to recognition of phases with particularly low resemblance of visual features or low procedural overlap. The heatmaps (Fig. 5) clearly illustrate that TEsoNet shows tendencies of confusing dissection phases (“Dissection Gastrocolic Ligament” and “Dissection Gastrohepatic Ligament”), which may be due to indistinct visual features, such as the anatomy of the ligaments or use of identical tools. And accuracy in recognition of shorter phases with low procedural overlap between the two operations appears to be negatively impacted by introduction of an unrelated procedure due to relative underrepresentation in the training data.

Whilst these fundamental differences between the two procedures will be hard to overcome, homogenous data attributes will facilitate TL. Differences in data acquisition and preprocessing may account for different perception of the data by ML algorithms. In our case, the two datasets were acquired in two different high-volume centers, and exhibited vastly different surgical traits (bariatric vs. oncological surgery, operating surgeons and teams, camera view, port placement and instruments, etc.). Moreover, divergent approaches to temporal annotation of surgical video data, due to the lack of standardized protocols [23, 24], impacts knowledge transfer. A more complex surgical workflow requires more granular annotation, which in turn results in higher clinical relevance [25]. Unified definitions for start and endpoints of surgical phases are necessary for congruent temporal annotations across procedures. Annotation guidelines have to account for more granular steps and actions within the phases to be applicable to complex procedures. In Esophagectomy for example, the “Hiatal Dissection” may be further divided into the dissection of the left and right Crus of the Diaphragm, and during “Dissection of the Gastrocolic Ligament” the individual quadrants along the greater curvature and the retro gastric space could be separately annotated.

TL for phase recognition was first performed by Neimark et al. [13]. The group demonstrated high accuracy when pretraining different model architectures on laparoscopic cholecystectomy and then evaluating performances on sleeve gastrectomy, right hemicolectomy, and appendectomy. This first attempt emphasized the need to explore different conditions under which TL can succeed. Besides low data quantities for training, other ML parameters such as training weights, pretraining methodology, and network architecture should be explored. In contrast to our work, Neimark’s focus was on overlap between the procedures with respect to broad surgical actions, like “adhesiolysis”, “preparation”, and “inspection”. However, this approach to annotating surgical phases lacks information about semantic content and temporal dependencies. Additionally, the used datasets were significantly larger in size, which raises the question of how much data is needed to transfer existing methodology to new procedures. Our goal was to investigate the performance of TL on clinically relevant, hard-to-obtain procedures such as IL-Esophagectomy, in low data conditions. Furthermore, we considered the relative contribution of source and target procedure on phase recognition accuracy. Expectedly, the linear workflow of Sleeve Gastrectomy was more detrimental to transferal model performance than the spatial characteristics of different anatomic landmarks in Esophagectomy.

Overall our work clearly supports selective TL for phases with high procedural overlap. Analogous to Hu et al.’s Frankenstein approach [26] to face recognition, who puzzled different subcomponents of faces together to generate new samples, we propose to leverage multiple operations for phase-specific TL. In the laparoscopic part of IL-Esophagectomy, information about dissection phases could be extracted from Nissen Fundoplicatio or Gastric Bypass to boost model performance on recognition of “Hiatal Dissection”. Similarly, phases covering clipping of tubular structures, such as “Clipping and Division of Left Gastric Artery” may be extrapolated from procedures like Laparoscopic Cholecystectomy. Leveraging overlapping temporal and visual features from a pool of multiple related procedures may lead to the generation of a diverse data catalog. Furthermore training on a wide array of surgical procedures, exhibiting various data characteristics may significantly benefit surgical AI by increasing data availability and diversity, hence promoting data efficiency.

## Conclusion

We demonstrate that phase recognition in less standardized, highly complex surgical procedures with a disruptive temporal workflow, such as Esophagectomy, is feasible using an established model structure. Our concise and clinically relevant annotations of the surgical phases in the laparoscopic part of IL-Esophagectomy can serve as a starting point for more granular definitions of surgical steps and actions in the future. Furthermore, we show that Transfer Learning between two visually different upper gastrointestinal procedures achieves good phase recognition accuracy in phases with high procedural overlap, despite low data quantities. Additionally, co-training on both procedures can further augment the phase-specific recognition accuracy selectively for said phases. To conclude, knowledge transfer between related procedures can help to overcome the shortage of meaningful, annotated surgical video data of rare, high-stake procedures and promote data efficiency. Further exploration of ideal training parameters and prerequisites for TL is necessary. In order to exploit the true risk mitigation potential of surgical AI, coverage of highly complex procedures is essential.

## References

[1] Twinanda AP, Shehata S, Mutter D, Marescaux J, de Mathelin M, Padoy N (2017). EndoNet: a deep architecture for recognition tasks on laparoscopic videos. IEEE Trans Med Imaging.

[2] Ban Y, Rosman G, Eckhoff JA, Ward TM, Hashimoto DA, Kondo T (2022). Supr-Gan: surgical prediction GAN for event anticipation in laparoscopic and robotic surgery. IEEE Robotics and Autom Lett.

[3] Ward TM, Hashimoto DA, Ban Y, Rattner DW, Inoue H, Lillemoe KD (2021). Automated operative phase identification in peroral endoscopic myotomy. Surg Endosc.

[4] Hashimoto DA, Rosman G, Witkowski ER, Stafford C, Navarette-Welton AJ, Rattner DW (2019). Computer vision analysis of intraoperative video: automated recognition of operative steps in laparoscopic sleeve gastrectomy: automated recognition of operative steps in laparoscopic sleeve gastrectomy. Ann Surg.

[5] Choi B, Jo K, Choi S, Choi J (2017) Surgical-tools detection based on Convolutional Neural Network in laparoscopic robot-assisted surgery. 2017 39th Annual International Conference of the IEEE Engineering in Medicine and Biology Society (EMBC): 1756–1759 10.1109/EMBC.2017.8037183 29060227

[6] Sarikaya D, Corso JJ, Guru KA (2017). Detection and localization of robotic tools in robot-assisted surgery videos using deep neural networks for region proposal and detection. IEEE Trans Med Imaging.

[7] Bouget D, Allan M, Stoyanov D, Jannin P (2017). Vision-based and marker-less surgical tool detection and tracking: a review of the literature. Med Image Anal.

[8] Madani A, Namazi B, Altieri MS, Hashimoto DA, Rivera AM, Pucher PH (2020). Artificial intelligence for intraoperative guidance: using semantic segmentation to identify surgical anatomy during laparoscopic cholecystectomy. Ann Surg.

[9] Nwoye CI, Gonzalez C, Yu T, Mascagni P, Mutter D, Marescaux J et al (2020) Recognition of instrument-tissue interactions in endoscopic videos via action triplets. arXiv:2007.05405. http://arxiv.org/abs/2007.05405 10.1016/j.media.2022.102433 35398658

[10] Ban Y, Eckhoff JA, Ward TM, Hashimoto DA, Meireles OR, Rus D et al (2022) Concept graph neural networks for surgical video understanding. arXiv:2202.13402. http://arxiv.org/abs/2202.13402 10.1109/TMI.2023.3299518 37498757

[11] Maier-Hein L, Vedula SS, Speidel S, Navab N, Kikinis R, Park A (2017). Surgical data science for next-generation interventions. Nat Biomed Eng.

[12] Lalys F, Jannin P (2014). Surgical process modelling: a review. Int J Comput Assist Radiol Surg.

[13] Neimark D, Bar O, Zohar M, Hager GD, Asselmann D (2021) “Train one, Classify one, Teach one” - Cross-surgery transfer learning for surgical step recognition. arXiv:2102.12308. http://arxiv.org/abs/2102.12308

[14] Jafari MD, Halabi WJ, Smith BR, Nguyen VQ, Phelan MJ, Stamos MJ (2013). A decade analysis of trends and outcomes of partial versus total esophagectomy in the United States. Ann Surg.

[15] Low DE, Allum W, De Manzoni G, Ferri L, Immanuel A, Kuppusamy M (2019). Guidelines for perioperative care in esophagectomy: enhanced recovery after surgery (ERAS®) society recommendations. World J Surg.

[16] Clark JM, Boffa DJ, Meguid RA, Brown LM, Cooke DT (2019). Regionalization of esophagectomy: where are we now?. J Thorac Dis.

[17] Meireles OR, Rosman G, Altieri MS, Carin L, Hager G, Madani A (2021). SAGES consensus recommendations on an annotation framework for surgical video. Surg Endosc.

[18] Farahani A, Pourshojae B, Rasheed K, Arabnia HR (2021) A concise review of transfer learning. arXiv:2104.02144. http://arxiv.org/abs/2104.02144

[19] Fuchs HF, Müller DT, Leers JM, Schröder W, Bruns CJ (2019). Modular step-up approach to robot-assisted transthoracic esophagectomy-experience of a German high volume center. Transl Gastroenterol Hepatol.

[20] Lin T-Y, Goyal P, Girshick R, He K, Dollár P (2017) Focal loss for dense object detection. arXiv:1708.02002. http://arxiv.org/abs/1708.02002 10.1109/TPAMI.2018.2858826 30040631

[21] Volkov M, Hashimoto DA, Rosman G, Meireles OR, Rus D (2017) Machine learning and coresets for automated real-time video segmentation of laparoscopic and robot-assisted surgery. 2017 IEEE International Conference on Robotics and Automation (ICRA): 754–759.

[22] Hamet P, Tremblay J (2017). Artificial intelligence in medicine. Metabolism.

[23] Nowak S, Rüger S (2010) How reliable are annotations via crowdsourcing: a study about inter-annotator agreement for multi-label image annotation. Proceedings of the international conference on Multimedia information retrieval: 557–566. New York, NY, USA: Association for Computing Machinery

[24] Ward TM, Fer DM, Ban Y, Rosman G, Meireles OR, Hashimoto DA (2021). Challenges in surgical video annotation. Comput Assist Surg (Abingdon).

[25] Garrow CR, Kowalewski KF, Li L, Wagner M, Schmidt MW, Engelhardt S (2021). Machine learning for surgical phase recognition: a systematic review. Ann Surg.

[26] Guosheng Hu, Peng X, Yang Y, Hospedales TM, Verbeek J (2018). Frankenstein: learning deep face representations using small data. IEEE Trans Image Process.

